# Evaluation of the Effects of Monosodium Glutamate Overconsumption on the Functions of the Liver, Kidney, and Heart of Male Rats: The Involvement of Dyslipidemia, Oxidative Stress, and Inflammatory Responses

**DOI:** 10.3390/jox15030064

**Published:** 2025-04-29

**Authors:** Heba M. Abdou, Amel H. El-Gendy, Rania Gaber Aly, Mekky M. Abouzied, Heba M. Eltahir, Sultan S. Al thagfan, Saber M. Eweda

**Affiliations:** 1 Zoology Department, Faculty of Science, Alexandria University, Alexandria 21515, Egypt; dr.heba_abdou3000@yahoo.com (H.M.A.); amel.hosny@alexu.edu.eg (A.H.E.-G.); 2 Department of Pathology, Faculty of Medicine, Alexandria University, Alexandria 21515, Egypt; rgm2006isa@yahoo.com; 3 Department of Biochemistry, Faculty of Pharmacy, Minia University, Minia 61516, Egypt; mabouzied@taibahu.edu.sa; 4 Department of Pharmacology and Toxicology (Biochemistry Subdivision), College of Pharmacy, Taibah University, Madinah 42353, Saudi Arabia; htahir@taibahu.edu.sa; 5 Department of Clinical and Hospital Pharmacy, College of Pharmacy, Taibah University, Madinah 42353, Saudi Arabia; salthagfan@gmail.com; 6 Biochemistry Department, Faculty of Science, Alexandria University, Alexandria 21515, Egypt; 7 Department of Clinical Laboratory Sciences, College of Applied Medical Sciences, Taibah University, Madinah 42353, Saudi Arabia

**Keywords:** monosodium glutamate, hepatotoxicity, renal toxicity, cardiac toxicity, oxidative stress, inflammation

## Abstract

The excessive intake of monosodium glutamate (MSG) increases its cellular levels in different organs and induces organ toxicity. The current study aims to investigate the metabolic changes and possible causes of hepatic, renal, and cardiac toxicity induced by MSG overconsumption. Thirty adult male rats were randomly allocated into five groups: control, MSG0.8, MSG1, MSG2, and MSG3, which were orally treated with a daily oral dose of saline, 0.8, 1, 2, and 3 g MSG/kg BW, respectively, for eight weeks. The hepatic, renal, and cardiac biochemical markers; lipid profile; glucose; electrolytes; iNOS; α-KGD; oxidative stress; and inflammatory markers were investigated. The histopathological examination of hepatic and renal tissues was also performed. The results revealed MSG-induced hepato-renal and cardiac toxicity, as indicated by the changes in the biochemical markers and tissue architecture of these organs. The toxicity is observed in the form of dyslipidemia, oxidative stress (increased MDA and NO and decreased GSH, SOD, CAT, and GST), and inflammatory responses (increased TNF-α and IL-6). The histopathological changes in liver and kidney architecture confirmed the obtained results. In conclusion, the MSG-induced hepatic, renal, and cardiac toxicity was dose-dependent, and awareness should be raised about the side effects of the overconsumption of MSG.

## 1. Introduction

The modern changes in lifestyle, the various marketing strategies, in addition to many other factors, resulted in an increased consumption of processed foods, an attitude that has been linked to various adverse effects on human health. This can be attributed to the excessive use of flavor enhancers, such as monosodium glutamate (C_15_H_8_NO_4_Na, MSG), during food processing [1]. MSG is a highly water-soluble, white, odorless, and colorless monohydrate crystalline powder. It is a sodium salt of the nonessential amino acid glutamic acid, containing 78% glutamic acid and 22% sodium and water [2]. MSG was first patented in 1908 by the Japanese chemist Kikunae Ikeda, who recognized glutamate as the key to the unique taste of seaweed and neutralized it with sodium to improve its solubility, producing MSG [3]. The characteristic flavor of MSG results from the activation of specific taste receptors on the tongue’s taste buds, known as umami receptors, which are responsible for transmitting taste signals to the brain to perceive this taste [4]. MSG improves the palatability of food by enhancing the umami taste [5]. There is no recommended daily dosage for MSG, and it is regarded as a safe food additive for human consumption. The Joint FAO/WHO Expert Committee on Food Additives evaluated the safety of glutamate and its salt in 1971, 1974, and 1987 and allocated it an “acceptable daily intake (ADI) not specified” [3]. However, the European Food Safety Authority (EFSA) re-evaluated the safety of MSG in 2017 and set an “acceptable daily intake” of 30 mg/kg BW [6]. Soups, chips and snack foods, frozen meals, and seasoning blends are the major sources of MSG.

Despite being considered a safe food additive, some research groups described the overconsumption of MSG as being associated with numerous pathological problems, such as neurological, hepatic, renal, and retinal disorders [7]. Recently, preclinical and clinical studies revealed that MSG may contribute to cardiovascular disorders, renal and hepatic toxicity, neurotoxicity, and metabolic syndrome [8]. Other reported side effects of the excessive use of MSG include headaches, flushing, weakness, and numbness, with exacerbation of other symptoms, such as dermatitis, urticaria, ventricular arrhythmia, and abdominal discomfort [9]. Other reports linked the consumption of MSG to decreased testosterone concentrations, testicular hemorrhage, and a dose-dependent increase in abnormal sperm morphology [10]. The prolonged intake of food containing MSG in animals and humans was shown to induce oxidative stress in the liver, pancreas, kidney, nervous system, and endocrine system [11]. A research group showed that every 1 g increase in the amount of consumed MSG increased the risk of metabolic syndrome or the development of an overweight condition, regardless of the level of physical activity [12].

To date, the adverse effects of consuming MSG on human health are still controversial due to the lack of known safe use limits. However, an increasing amount of experimental evidence reveals that the regular and overconsumption of MSG in fast foods can be associated with systemic disorders and alteration of signaling pathways in humans and experimental animals [13].

The current study was designed to investigate the impact of different concentrations of MSG on the hepatic, cardiac, and renal integrity of male albino rats, as well as identifying the toxic MSG dose on these organs based on oxidative stress markers, histopathology, and inflammatory response. The data obtained could be used to help set a preliminary safe maximum dose for daily MSG consumption.

## 2. Materials and Methods

### 2.1. Chemicals and Reagents

Anhydrous monosodium glutamate (99% purity) was obtained from Loba Chemie for laboratory reagents and fine chemicals (Mumbai, India). All chemicals and reagents were of analytical grade and purchased from Sigma Aldrich (St. Louis, MO, USA). Kits used in the study were purchased from the local and international markets.

### 2.2. Experimental Design, Sampling and Tissues Preparation

Adult male Wistar rats (about 12 weeks old, 150–170 g) were purchased from the Faculty of Medicine, Alexandria University, Egypt. They were housed in stainless-steel cages under standard conditions (temperature 25 °C, relative humidity of 50–60%, and 12-h light/dark cycle) during the experiment. Rats had free access to a standard basal diet and tap water ad libitum. The experimental protocol was approved by Alexandria University Institutional Animal Care and Use Committee (ALEXU-IACUC), a member of the International Council for Laboratory Animal Science (ICLAS) (Approval number: AU 04230530301). After two weeks of acclimatization, rats were randomly allocated into five groups, each of 6 rats (control group and four MSG-treated groups: MSG 0.8, MSG 1, MSG 2, and MSG 3). The control group received a daily oral dose of saline. The animals of MSG-treated groups received an oral MSG dose of 0.8, 1, 2, or 3 mg/g BW/day, respectively, for 8 weeks at consistent times. The MSG doses used were selected based on the doses reported by previous studies and the LD50 of MSG [10,14].

By the end of the experimental period, rats were fasted for 12 h, and then they were sacrificed under deep anesthesia via an intraperitoneal injection of 100 mg/kg ketamine and 20 mg/kg xylazine. Blood samples collected from the animals were centrifuged at 4000 rpm for 15 min after allowing them to coagulate for 30 min at room temperature. The separated serum samples were stored at −80 °C for further biochemical analysis. The liver, kidney, and heart were immediately collected after death, briefly rinsed in ice-cold saline and prepared either for histopathological investigation by fixing parts of the organs in 10% formalin or homogenized (10% *w*/*v*) in 50 mM cold potassium phosphate (pH 7.4). Tissue homogenates were centrifuged at 12,000 rpm at 4 °C for 15 min and the supernatants were stored at −80 °C for assessing the oxidative stress and inflammatory markers.

### 2.3. Serum Biochemical Measurements

For assessment of the tested biochemical parameters, commercially available kits were utilized according to the manufacturer’s instructions.

The activities of aspartate aminotransferase (AST), alanine aminotransferase (ALT), γ-glutamyl transferase (GGT), alkaline phosphatase (ALP), lactate dehydrogenase (LDH), and creatine kinase-MB (CK-MB) in the serum of rats were assayed spectrophotometrically using Bio-diagnostic and Research Company kits (Bio-diagnostic and Research Company, Dokki, Giza, Egypt).

The serum concentration of total proteins, albumin, bilirubin, urea, creatinine, uric acid, calcium, and sodium was determined calorimetrically (Spectrum Company kits, Egypt). The serum fasting blood glucose (FBG) was determined by Randox colorimetric reagent kits (Antrim, UK).

Commercial colorimetric kits from Boehringer Mannheim (Germany) were used for the determination of serum triglyceride (TG) and total cholesterol (TC) concentrations. The high-density lipoprotein cholesterol fraction was measured according to the procedure of Lopes-Virella [15] and the “Friedewald equation” [LDL-C = TC-(HDL-C + 1/5 TGs)] was used to calculate the concentration of low-density lipoprotein-cholesterol fraction [16]. The results were expressed in mg/dL.

The total creatine phosphokinase was measured using an ELISA kit (My BioSource, San Diego, CA, USA, Cat number: MBS1608443) according to the manufacturer’s protocol. The α-ketoglutarate dehydrogenase (α-KGD) activity was measured using Abcam’s colorimetric assay kit (ab185440, Waltham, MA, USA). The inducible nitric oxide synthase (iNOS) was assayed using a double-antibody sandwich ELISA technique. The intensity of the yellow color produced is positively correlated with the amount of enzyme in the sample. A calibration curve was constructed and used to calculate the concentration of iNOS in the tested samples.

### 2.4. Assessment of Oxidative Stress and Antioxidant Markers in the Liver, Kidney and Heart Tissues

The concentrations of glutathione (GSH), nitric oxide (NO), and malonaldehyde (MDA) in tissue homogenates were assessed using commercially available kits (Biodiagnostic and Research Company, Egypt) according to the manufacturer’s instructions. Catalase (CAT) activity was assayed according to the protocol described by Bergmeyer (2012) [17]. The activity of superoxide dismutase (SOD) was determined by the method of Fridovich (1989) [18]. Glutathione-S-transferase (GSH) activity was assayed in tissue homogenates according to the method of Habig et al. (1974) using p-nitrobenzyl chloride as a substrate [19].

### 2.5. Assessment of Inflammatory Markers in the Liver, Kidney and Heart Tissues

The tissue level of interleukin-6 (IL-6) and tumor necrosis factor-α (TNF-α) was assessed in hepatic, renal, and cardiac tissue homogenates using a solid phase sandwich ELISA protocol based on the method described previously using commercially available kits according to the manufacturer’s instructions [20,21].

### 2.6. Histopathological Investigations of Liver and Kidney

Parts of the liver and kidney fixed in 10% neutral buffered formalin were dehydrated in a series of increasing alcohol concentrations, then cleared with xylene, and finally embedded in paraffin. Five-micrometre thick sections were cut on a microtome, and the sections were deparaffinized then stained with hematoxylin and eosin (H&E) for histopathological examination [22].

### 2.7. Statistical Analysis

Results are expressed as mean ± standard error (SE). For the statistical analysis, a Social Sciences software package (SPSS, version 8.0, Chicago, IL, USA) was used. One-way analysis of variance (ANOVA) was used for determining the differences between experimental groups. A post hoc test (Tukey–Kramer test) was used for statistical comparison between groups. The values were statistically significant at *p* ≤ 0.05.

## 3. Results

### 3.1. Monosodium Glutamate Induces Changes in the Biochemical Markers of Liver, Kidney, and Heart Functions

**Figure 1:**
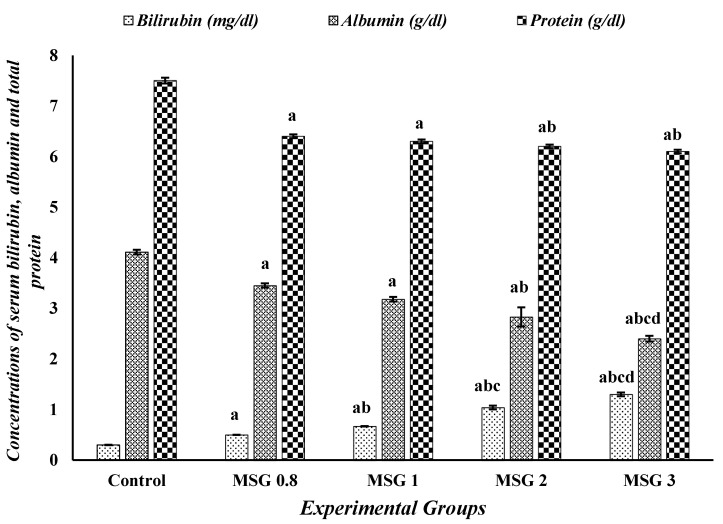
Effect of treatments on the serum concentrations of bilirubin (mg/dL), albumin (g/dL) and total protein (g/dL) among different experimental groups. All data were analyzed using one-way ANOVA followed by the post hoc (Tukey) test. Values are expressed as the means of 10 rats ± SE. ^a^ Significant (*p* < 0.05) difference of all groups compared to the control group, ^b^ significant (*p* < 0.05) difference of treated groups compared to the MSG 0.8 group, ^c^ significant (*p* < 0.05) difference compared to the MSG 1 group, ^d^ significant (*p* < 0.05) difference compared to the MSG 2 group.

The activities of AST, ALT, GGT, and ALP, as well as the serum levels of bilirubin, albumin, and total proteins of different experimental groups, are depicted in Table 1 and Figure 1. The AST, ALT, GGT, and ALP activities and bilirubin level were significantly increased (*p* ˂ 0.05) in all treated groups compared to control, while the concentration of serum albumin and total protein showed a significant (*p* ˂ 0.05) reduction in the treated groups compared to control. The effect on enzyme activities and bilirubin level was dose-dependent, where the highest effect was observed in the MSG 3 group, while the lowest effect was observed in MSG 0.8.

A significant (*p* ˂ 0.05) elevation was observed on the levels of renal function markers including serum urea, creatinine, and uric acid in all treated groups compared to the control group. Similar to the previous observation, the effect on these parameters was dose dependent as the MSG concentration increased, reflecting various degrees of renal damage (Table 2).

**Figure 2:**
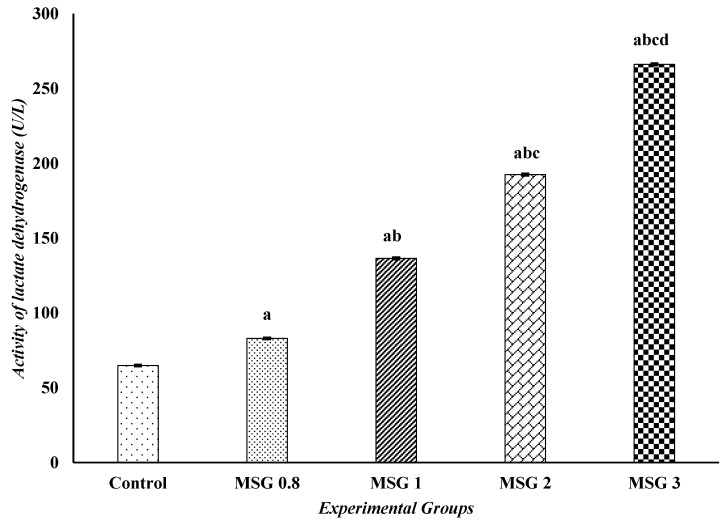
Effect of treatments on the activities of lactate dehydrogenase (U/L) among different experimental groups. All data were analyzed using one-way ANOVA followed by the post hoc (Tukey) test. Values are expressed as the means of 10 rats ± SE. ^a^ Significant (*p* < 0.05) difference of all groups compared to the control group, ^b^ significant (*p* < 0.05) difference of treated groups compared to the MSG 0.8 group, ^c^ significant (*p* < 0.05) difference compared to the MSG 1 group, ^d^ significant (*p* < 0.05) difference compared to the MSG 2 group.

**Figure 3:**
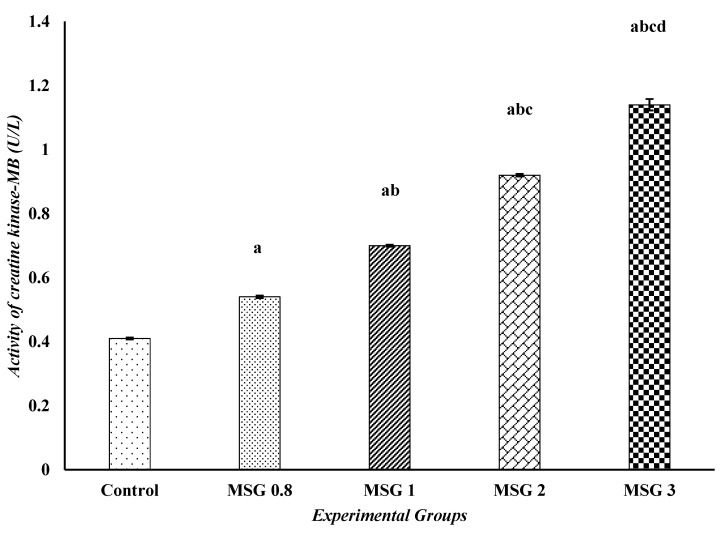
Effect of treatments on the activities of creatine kinase-MB (U/L) among different experimental groups. All data were analyzed using one-way ANOVA followed by the post hoc (Tukey) test. Values are expressed as the means of 10 rats ± SE. ^a^ Significant (*p* < 0.05) difference of all groups compared to the control group, ^b^ significant (*p* < 0.05) difference of treated groups compared to the MSG 0.8 group, ^c^ significant (*p* < 0.05) difference compared to the MSG 1 group, ^d^ significant (*p* < 0.05) difference compared to the MSG 2 group.

The effect on heart function parameters, LDH and CK-MB activities, is presented in Figure 2 and Figure 3. The results revealed a significant (*p* ˂ 0.05) elevation in the activities of LDH and CK-MB in the MSG 0.8, 1, 2, and 3 groups compared to control. It is to be noted that the effect of MSG here is also dose-dependent, where the highest dose (MSG 3 group) showed significantly higher levels of heart function parameters compared to the lower doses (*p* ˂ 0.05).

### 3.2. Monosodium Glutamate Induces a State of Dyslipidemia

Table 3 represents the effect of MSG doses on the lipid profile. The pairwise comparison revealed a significant (*p* ˂ 0.05) increase in the serum levels of TC, TG, and LDL-C and a significant decrease in the HDL-C in the MSG-treated groups compared to control, following a dose-dependent manner. Treatment with 3 mg/g MSG resulted in significant effects on all lipid profile parameters compared to the lower doses of MSG.

### 3.3. Monosodium Glutamate Induces Hyperglycemia and Electrolytes Imbalance

**Figure 4:**
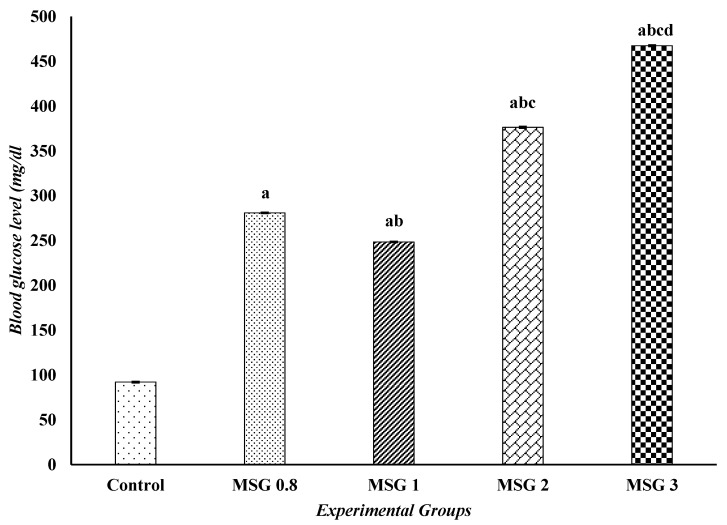
Effect of treatments on the blood glucose levels (mg/dL) among different experimental groups. All data were analyzed using one-way ANOVA followed by the post hoc (Tukey) test. Values are expressed as the means of 10 rats ± SE. ^a^ significant (*p* < 0.05) difference of all groups compared to the control group, ^b^ Significant (*p* < 0.05) difference of treated groups compared to the MSG 0.8 group, ^c^ significant (*p* < 0.05) difference compared to the MSG 1 group, ^d^ significant (*p* < 0.05) difference compared to the MSG 2 group.

The one-way ANOVA showed a significant (*p* < 0.05) elevation in the blood glucose levels of all MSG-treated groups compared to the control group. On the other hand, there was a significant (*p* < 0.05) decrease in the serum levels of calcium and sodium in all treated groups compared to the control group. The changes in glucose, calcium, and sodium concentrations are dose-dependent (Table 2 and Figure 4).

### 3.4. Monosodium Glutamate Induces Changes in the Activities of iNOS, CPK, and α-KGD

**Figure 5:**
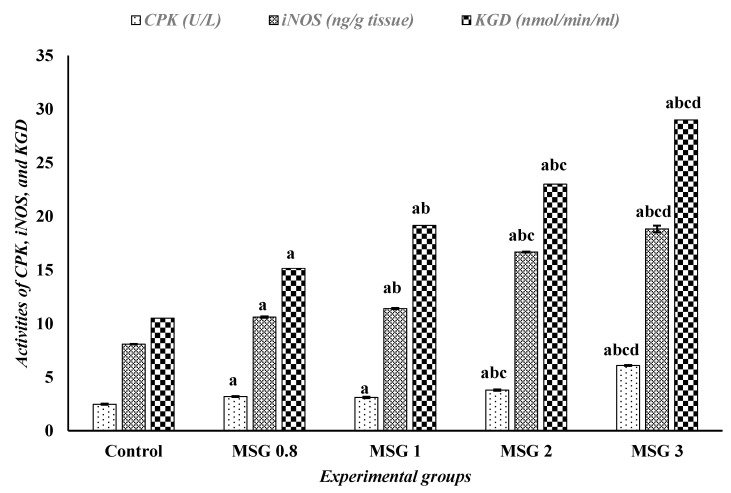
Effect of treatments on the activities of CPK (U/L), iNOS (ng/g tissue) and KGD (nmol/min/mL) among different experimental groups. All data were analyzed using one-way ANOVA followed by the post hoc (Tukey) test. Values are expressed as the means of 10 rats ± SE. ^a^ Significant (*p* < 0.05) difference of all groups compared to the control group, ^b^ significant (*p* < 0.05) difference of treated groups compared to the MSG 0.8 group, ^c^ significant (*p* < 0.05) difference compared to the MSG 1 group, ^d^ significant (*p* < 0.05) difference compared to the MSG 2 group. CPK; creatine phosphokinase, iNOS; inducible nitric oxide synthase, KGD; ketoglutarate dehydrogenase.

As shown in Figure 5, the activities of iNOS, CPK, and α-KGD were significantly increased (*p* < 0.05) in all treated groups compared to the control group in a dose-dependent fashion.

### 3.5. Monosodium Glutamate Induces Hepatic, Renal, and Cardiac Oxidative Stress

The daily consumption of MSG at different doses for eight weeks induced oxidative stress in the liver, kidney, and heart tissues. Results of the present study showed a significant increase (*p* < 0.05) in the concentrations of MDA and NO in hepatic, renal, and cardiac tissues of the MSG-treated groups compared to control. In contrast, the endogenous antioxidant capacity represented by GSH levels and the activities of CAT, SOD, and GST in the liver, kidney, and heart of such treated groups was significantly (*p* < 0.05) depleted compared to the control groups in a dose-dependent manner. MSG 0.8 showed mild oxidative stress, which increased in a dose-dependent manner as the concentration of MSG increased (Table 4, Table 5 and Table 6).

### 3.6. Effect of Monosodium Glutamate on Hepatic, Renal, and Cardiac Inflammatory Response

**Figure 6:**
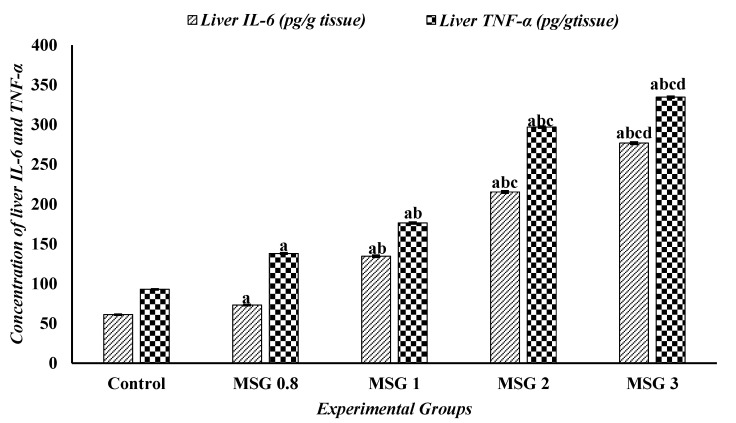
Effect of treatments on the concentrations of liver IL-6 (pg/g tissue) and TNF-α (pg/g tissue) among different experimental groups. All data were analyzed using one-way ANOVA followed by the post hoc (Tukey) test. Values are expressed as the means of 10 rats ± SE. ^a^ Significant (*p* < 0.05) difference of all groups compared to the control group, ^b^ significant (*p* < 0.05) difference compared to the MSG 0.8 group, ^c^ significant (*p* < 0.05) difference compared to the MSG 1 group, ^d^ significant (*p* < 0.05) difference compared to the MSG 2 group. IL-6; interlukin-6 and TNF-α; tumor necrosis factor-α.

**Figure 7:**
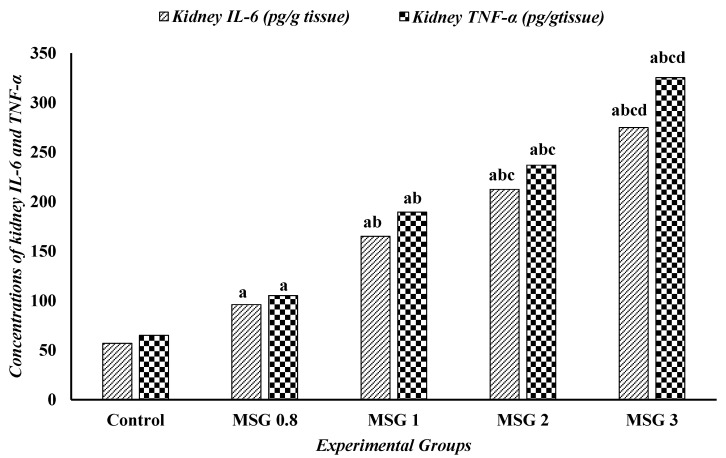
Effect of treatments on the concentrations of kidney IL-6 (pg/g tissue) and TNF-α (pg/g tissue) among different experimental groups. All data were analyzed using one-way ANOVA followed by the post hoc (Tukey) test. Values are expressed as the means of 10 rats ± SE. ^a^ significant (*p* < 0.05) difference of all groups compared to the control group, ^b^ significant (*p* < 0.05) difference compared to the MSG 0.8 group, ^c^ significant (*p* < 0.05) difference compared to the MSG 1 group, ^d^ significant (*p* < 0.05) difference compared to the MSG 2 group. IL-6; interlukin-6 and TNF-α; tumor necrosis factor-α.

**Figure 8:**
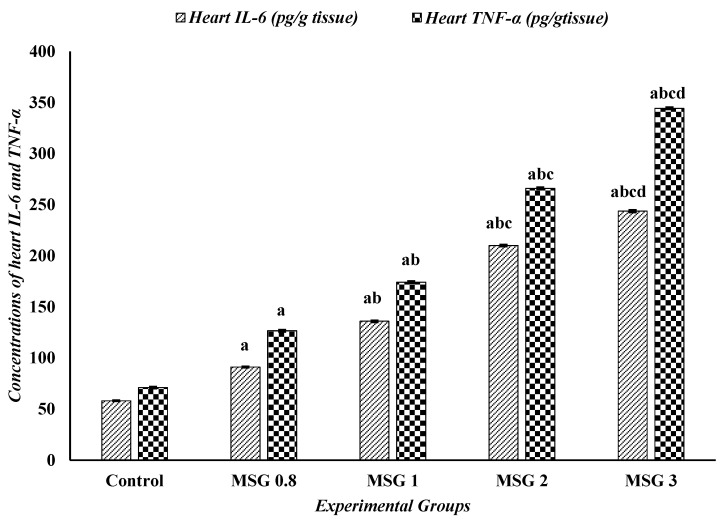
Effect of treatments on the concentrations of heart IL-6 (pg/g tissue) and TNF-α (pg/g tissue) among different experimental groups. All data were analyzed using one-way ANOVA followed by the post hoc (Tukey) test. Values are expressed as the means of 10 rats ± SE. ^a^ Significant (*p* < 0.05) difference of all groups compared to the control group, ^b^ significant (*p* < 0.05) difference compared to the MSG 0.8 group, ^c^ significant (*p* < 0.05) difference compared to the MSG 1 group, ^d^ significant (*p* < 0.05) difference compared to the MSG 2 group. IL-6; interlukin-6 and TNF-α; tumor necrosis factor-α.

The one-way ANOVA followed by Tukey’s test revealed a significant elevation (*p* ≤ 0.5) in the levels of TNF-α and IL-6 in the liver, kidney, and heart of MSG-treated groups compared to control. The proinflammatory markers, TNF-α and IL-6, significantly increased (*p* ≤ 0:05) in a dose-dependent manner in the three organ tissues (Figure 6, Figure 7 and Figure 8).

### 3.7. Monosodium Glutamate Elicited Histopathological Alterations in Hepatic and Renal Tissues

**Figure 9:**
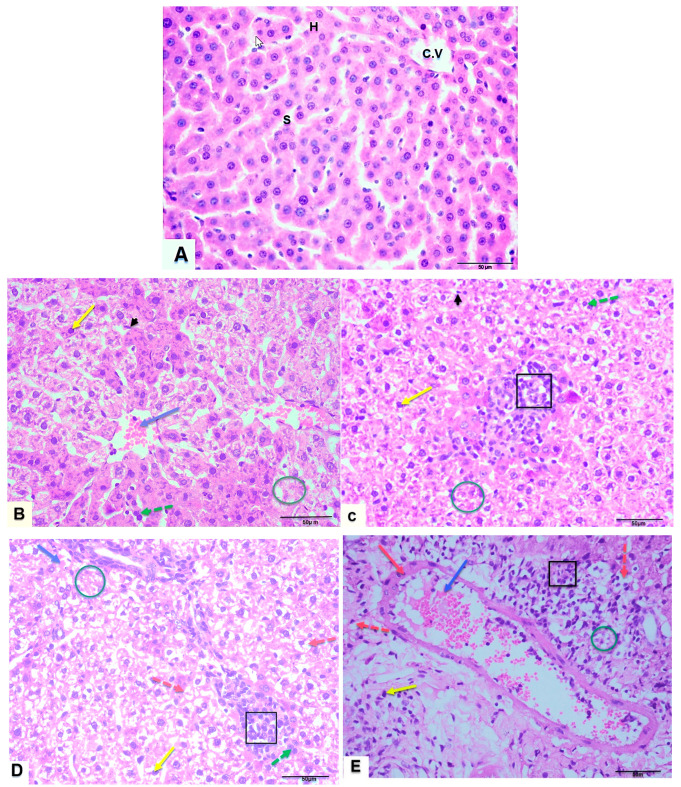
Photomicrographs of T.S in the liver tissues of control rats (**A**) and MSG-treated rats with different doses (**B**–**E**), respectively. Control rats (**A**) showing normal hepatocyte structure with normal vesiculated nuclei (H), central vein (C.V) and blood sinusoids (s). While different experimental groups—MSG 0.8-treated rats (**B**), MSG 1-treated rats (**C**), MSG 2-treated rats (**D**), and MSG 3-treated rats (**E**)—showed loss of the normal hepatocyte architecture; vacuoles (red dotted arrow), degeneration of hepatocytes (green circle), more Kupffer cells (black head arrow), pyknotic nuclei (yellow arrow), inflammatory infiltrate (black square), hemorrhage (blue arrow), binucleated hepatocytes (green dotted arrow), and dilatation of blood vessels (red arrow) (H & E stain, ×400).

MSG prompted pronounced histopathological aberrations in hepatic and renal tissues. Microscopic evaluation of liver tissue in control rats disclosed well-preserved hepatocyte cytoarchitecture, featuring condensed nuclei, central veins, and organized blood sinusoids. In contrast, liver sections from MSG-administered rats demonstrated notable structural disorganization, including hepatic cell vacuolation, deterioration, amplified Kupffer cell proliferation, karyorrhectic nuclei, inflammatory cell infiltration, extravasation, binucleated hepatocytes, and dilated vasculature. The most extensive hepatic aberrations were observed in the MSG 3 cohort (Figure 9A–E).

**Figure 10:**
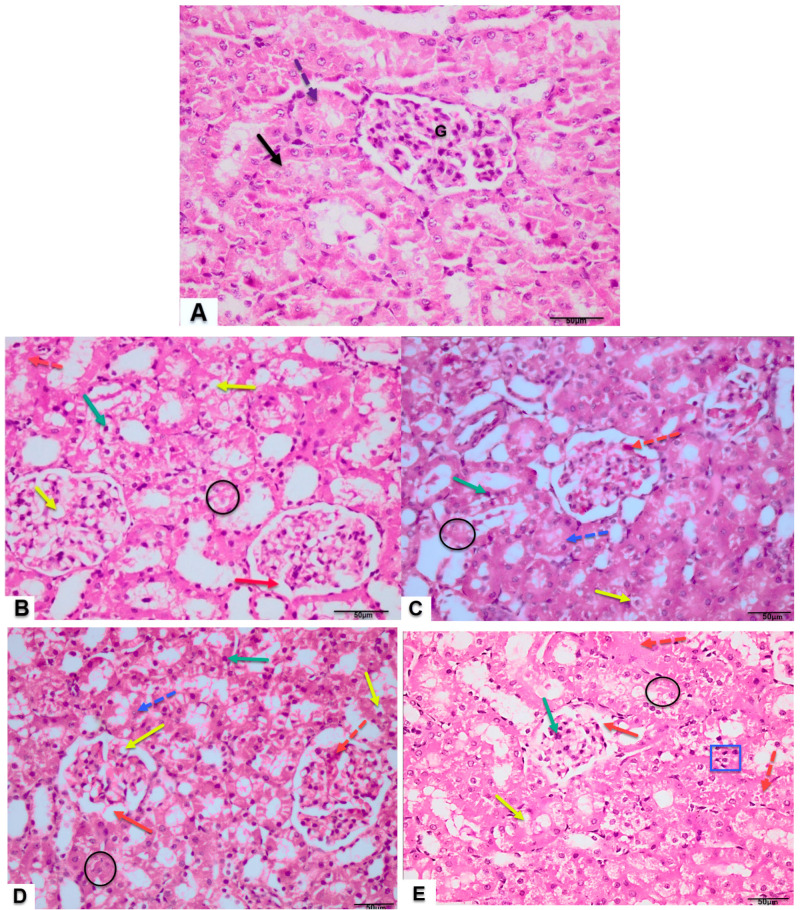
Photomicrographs of T.S in kidney tissue of control rats and MSG-treated rats with different doses (**B**–**E**), respectively. Control rats (**A**) showing normal cortical architecture with normal glomerulus (G), proximal convoluted tubules (dotted arrow), and distal convoluted tubules (black arrow). While different experimental groups—MSG 0.8-treated rats (**B**), MSG 1-treated rats (**C**), MSG 2-treated rats (**D**) and MSG 3-treated rats (**E**)—showed shrunken glomeruli with capsular space (red arrow), degenerative changes in the tubular cells (black circle), pyknotic nuclei (green arrow), cloudy swelling (blue dotted arrow) and hemorrhage (red dotted arrow), vacuolization (yellow arrow), and inflammatory infiltrate (blue square) (H & E ×400).

Renal histopathological assessment across the experimental cohorts (Figure 10A–E) revealed preserved cortical organization with intact glomeruli, proximal tubules, and distal tubules in healthy control sections. However, kidneys from MSG-administered cohorts displayed contracted glomeruli with widened Bowman’s space, tubular degeneration, karyorrhectic nuclei, hydropic degeneration, hemorrhagic regions, vacuolation, and leukocytic infiltration. It is to be noted that the severity of tissue damage was proportional to the dose of MSG, where the strongest effect was observed in animals treated with the highest dose (MSG 3).

## 4. Discussion

Monosodium glutamate poses a worldwide concern due to its widespread use as a flavor and taste enhancer and food additive. Many processed foods, such as sauces, mixed condiments, snacks, and soups, contain MSG under the code E621 [23]. Notably, the daily consumption of MSG was reported to induce various adverse effects in different organs, such as the liver, heart, brain, kidney, and thymus [24]. Based on the ADI of MSG, which was assigned by the EFSA as 30 mg/Kg BW [7], the current study attempts to use doses of MSG much higher than the reported acceptable daily limit and, at the same time, much lower than its LD_50_ to evaluate the adverse effects of MSG overconsumption.

The current study revealed that MSG in the tested doses induced hepatic, renal, and cardiac dysfunction through the induction of dyslipidemia, aggravation of oxidative stress via disruption of oxidant/antioxidant balance and increased the inflammatory reactions in these organs. These deleterious effects were dose-dependent and increased as the MSG concentration increased.

The different doses of MSG used in the present study led to impairment in the synthetic, conjugation, detoxification, and excretory functions of the liver and affected its cellular integrity, as observed by the decreased serum levels of albumin and total proteins, the increased serum bilirubin concentration, and the increased activities of serum AST, ALT, GGT, and ALP. Previous studies support the hepatic degenerative changes observed in the current study [5,25]. These are not the only reported pathological changes to the liver after MSG use, as it was reported by others that the overconsumption of MSG decreased the permeability of the hepatic cellular membrane in rats, resulting in the dilation of central hepatic veins, deformation of liver cells, and lysis of RBCs [26]. The decreased albumin synthesis and increased bilirubin and liver-specific enzymes in the blood were reported to be associated with hepatocellular damage [27].

Urea, creatinine, and uric acid are byproducts of protein, phosphocreatine, and nucleotide catabolism, respectively, and are eliminated from the body by the kidneys through urine. The serum levels of these markers are commonly used as indicators of renal function [28]. The current study showed that the different doses of MSG used have nephrotoxic effects, as indicated by the elevated serum levels of urea, creatinine, and uric acid, as well as the histopathological changes. These findings are consistence with the results from previous reports [1]. The increased serum levels of urea and creatinine in the treated animals indicate a decreased glomerular filtration rate and deterioration of renal functions.

As observed in the current work, the nephrotoxic effect of MSG disturbed the electrolyte balance, resulting in decreased serum levels of calcium and sodium in a dose-dependent manner. It has been reported that the renal toxicity of MSG can be attributed to its potential role in ROS generation, increased activity of α-KGD, and renal injury induced by increased phospholipase activity [29]. The increased MSG-induced ROS production exacerbates the damage to renal glomeruli and tubules [30].

In addition to hepatic and renal damage, MSG increased the serum activity of cardiac enzymes such as AST, LDH, and CPK, especially the heart-specific CK-MB fraction. Such elevation of cardiac enzymes indicates damage to cardiac tissue and increased permeability of myocardial cells. Our results are in line with Banerjee et al. (2021), who reported an increase in serum levels of cardiac damage markers, such as LDH and AST, in response to MSG administration [31]. Another report revealed that doses of MSG between 0.5–1.5 g/kg BW increased the probability of tachyarrhythmia in myocardial infarcted rats in a dose-dependent manner [32]. MSG-induced cardiac damage was reported to be stimulated via aggravation of oxidative stress, dyslipidemia, and necrosis of cardiomyocytes [33].

The use of MSG in the current study induced a condition of dyslipidemia. This can be attributed to the induced liver damage, as well as underlying metabolic disturbances, which represent a major risk factor for steatohepatitis and cardiovascular diseases, as reported earlier [34]. It is to be noted that hyperlipidemia depletes the endogenous antioxidant defense system. Such depletion of antioxidants and MSG-induced oxidative damage may explain the observed hepatic, renal, and cardiac damage.

MSG has been reported to reduce leptin, compromising the appetite regulation center and leading to hyperphagia and metabolic disturbances. These include an abnormal lipid profile, body weight gain, hyperglycemia, and insulin resistance [35]. Persistent hyperglycemia, as well as the activation of HMG CoA reductase, shifts glucose metabolism towards lipogenesis, resulting in hypercholesterolemia [36]. This explains the observed MSG-induced hyperglycemia and dyslipidemia, suggesting a possible defect in insulin sensitivity. Another possible explanation for disrupted glucose metabolism could be the alteration in the activity of pancreatic β-cells [37]. Overconsumption of MSG has been reported to increase the incidence of type 2 diabetes through decreased β-cell mass, increased ROS production, and fibrosis of pancreatic islets [34]. Others suggested that MSG could induce hyperglycemia by downregulating the levels of glucose transporter 4 (GluT-4) protein [38]. Furthermore, MSG-induced ROS production was suggested to oxidize thiol groups in insulin receptors, leading to insulin resistance, and inhibit TG uptake, resulting in hyperglycemia and hypertriglyceridemia [39].

The MSG-induced hepatic, renal, and cardiac damage may be attributed to the oxidative stress and inflammatory reaction resulting from overconsumption. In the current study, MSG induced the production of MDA and NO and reduced the levels of reduced glutathione and the activity of CAT, SOD, and GST in liver, kidney, and heart tissues. These findings add to the mounting evidence confirming MSG-induced ROS production, which aggravates peroxidation of membrane lipids and hence cellular damage [40]. GSH is a main component of the endogenous antioxidant defense system that acts as a scavenger for radicals and a stabilizer of membrane phospholipids. Depletion of GSH is usually associated with tissue damage that is correlated with the degree of GSH depletion [41]. Additionally, the current study revealed the elevation of pro-inflammatory mediators, IL-6 and TNF-α, in these organs in response to MSG administration. One possible reason for the increase in their levels could be the oxidative stress condition induced by MSG administration. Such an imbalance between ROS and endogenous antioxidants has been reported by a large number of studies to play a major role in inducing an inflammatory response within different organs and upregulating the levels of pro-inflammatory cytokines. Previous studies reported a significant increase in hepatic and renal lipid peroxidation as well as a decrease in endogenous antioxidants (GSH, CAT, SOD, GPx, and GST) after acute or chronic administration of 4 g MSG/kg [42,43]. Another study reported an increase in cardiac oxidative stress markers and increased AST, LDH, and ALT activities as well [31].

The inflammatory effect of MSG can be explained by its ability to induce oxidative stress and activate the nuclear factor kB (NFkB) pathway, resulting in transcriptional activation of downstream pro-inflammatory cytokines, e.g., TNF-α and IL-6 [44]. MGS-mediated ROS production has been shown to induce inflammation, as marked by the increased levels of pro-inflammatory cytokines, and cause liver fibrosis and cell death [41]. It is to be mentioned that oxidative modifications of proteins and lipids in heart tissue may induce weak contractility and myocardial infarction, resulting in sudden death [45].

One of the main factors that aggravates oxidative stress is the increased activity of NADPH oxidase, which represents a major source of superoxide anion and hydrogen peroxide in hepatic and non-hepatic tissues [46]. The overwhelming production of ROS depletes the endogenous antioxidants, and the activity of antioxidant enzymes is also reduced as they become cross-linked to MDA [47]. One of the possible ways by which MSG induces oxidative stress can be attributed to glutamate. This amino acid interacts with the NMDA (N-methyl-D-aspartate) receptor, leading to calcium influx and the activation of protein kinase and nitric oxide synthase with the subsequent generation of ROS [48]. This is confirmed by our findings, which revealed an increased activity of iNOS in all MSG-treated groups in a dose-dependent manner.

The MSG-induced activation of iNOS and the disruption of redox equilibrium due to excessive ROS production are key players in initiating an inflammatory response, inducing tissue damage, and increasing susceptibility to peroxidation and DNA damage [1].

The excessive amounts of glutamate in an MSG-rich diet also increase the activity of α-KGD, resulting in activation of NMDA receptor and acceleration of the Krebs cycle. This increases mitochondrial superoxide ion production and interferes with NADPH production, resulting in depletion of endogenous antioxidants, which leads to hepatotoxicity, nephrotoxicity, and cardiotoxicity [43]. This postulation was confirmed by the present study, which found that the activity of α-KGD markedly increased with the intake of MSG in a dose-dependent manner. Therefore, the increased activity of α-KGD plays an important role in the exacerbation of oxidative stress induced by MSG.

The hepatic and renal injuries induced by monosodium glutamate (MSG) were corroborated through histopathological evaluation. The histopathological alterations detected in the hepatic and renal tissues of the MSG-treated groups are consistent with the findings reported by multiple research groups, indicating the deleterious effects of MSG at the cellular level [5,49,50].

## 5. Conclusions

In conclusion, the excessive intake of MSG above the previously reported safe limit induced hepatic, renal, and cardiac damage. The study suggests that the observed organ damage induced by MSG can be attributed to dyslipidemia, oxidative stress, and induction of inflammation. The increased activities of α-ketoglutarate synthase and inducible nitric oxide synthase may be primary causes of the excessive production of ROS and the aggravation of oxidative stress. The findings of this study could be shared to raise awareness about the risk of MSG overconsumption.

## Figures and Tables

**Table 1:** Effects of different doses of monosodium glutamate on the activities of AST, ALT, GGT, and ALP in the serum of male rats.

Parameters	Experimental Groups				
	Control	MSG 0.8	MSG 1	MSG 2	MSG 3
Aspartate aminotransferase (AST) (U/L)	64.33 ± 0.881	98.50 ± 0.763 ^a^	118.66 ± 0.494 ^ab^	182.50 ± 0.763 ^abc^	241.50 ± 0.763 ^abcd^
Alanine aminotransferase (ALT) (U/L)	36.50 ± 0.763	86.50 ± 0.428 ^a^	96.00 ± 0.577 ^ab^	136.50 ± 0.763 ^abc^	204.16 ± 0.600 ^abcd^
γ-Glutamyl transferase (GGT) (U/L)	3.65 ± 0.042	6.00 ± 0.036 ^a^	12.46 ± 0.049 ^ab^	18.08 ± 0.047 ^abc^	20.20 ± 0.073 ^abcd^
Alkaline phosphatase (ALP) (U/L)	2.80 ± 0.057	3.80 ± 0.036 ^a^	3.96 ± 0.033 ^a^	4.38 ± 0.047 ^abc^	4.58 ± 0.047 ^abcd^

Values are expressed as means ± SE. Statistical significance was tested using ANOVA followed by post hoc Tukey’s HSD multiple comparison test. ^a^ The mean values are significantly different in comparison with the control group at *p* ≤ 0.05. ^b^ The mean values are significantly different in comparison with the MSG 0.8 group at *p* ≤ 0.05. ^c^ The mean values are significantly different in comparison with the MSG 1 group at *p* ≤ 0.05. ^d^ The mean values are significantly different in comparison with the MSG 2 group at *p* ≤ 0.05.

**Table 2:** Effects of different doses of monosodium glutamate on the concentrations of urea, creatinine, uric acid, calcium, and sodium in the serum of male rats.

Parameters	Experimental Groups				
	Control	MSG 0.8	MSG 1	MSG 2	MSG 3
Urea (mg/dL)	16.66± 0.666	27.50 ± 0.763 ^a^	32.16 ± 0.654 ^ab^	46.16 ± 0.477 ^abc^	54.66 ± 0.666 ^abcd^
Creatinine (mg/dL)	0.28 ± 0.016	0.46 ± 0.004 ^a^	0.67 ± 0.004 ^ab^	1.20 ± 0.036 ^abc^	1.78 ± 0.047 ^abcd^
Uric acid (mg/dL)	2.00 ± 0.036	2.51 ± 0.047 ^a^	3.31 ± 0.060 ^ab^	3.95 ± 0.042 ^abc^	4.65 ± 0.076 ^abcd^
Calcium (mg/dL)	10.31 ± 0.047	8.28 ± 0.060 ^a^	7.78 ± 0.047 ^ab^	7.38 ± 0.047 ^abc^	6.75 ± 0.042 ^abcd^
Sodium (mmol/L)	149.66 ± 0.557	136.83 ± 0.477 ^a^	132.66 ± 0.666 ^ab^	125.83 ± 0.600 ^abc^	118.00 ± 0.577 ^abcd^

Values are expressed as means ± SE. Statistical significance tested using ANOVA followed by post hoc Tukey’s HSD multiple comparison test. ^a^ The mean values are significantly different in comparison with the control group at *p* ≤ 0.05. ^b^ The mean values are significantly different in comparison with the MSG 0.8 group at *p* ≤ 0.05. ^c^ The mean values are significantly different in comparison with the MSG 1 group at *p* ≤ 0.05. ^d^ The mean values are significantly different in comparison with the MSG 2 group at *p* ≤ 0.05.

**Table 3:** Effect of different doses of monosodium glutamate on the serum lipid profile (TC, TG, LDL, and HDL) of male rats.

Parameters	Experimental Groups				
	Control	MSG 0.8	MSG 1	MSG 2	MSG 3
Total cholesterol (TC) (mg/dL)	88.16± 0.600	128.50 ± 0.428 ^a^	136.00 ± 0.365 ^ab^	169.00 ± 0.577 ^abc^	204.33 ± 0.954 ^abcd^
Triglycerides (TG) (mg/dL)	58.50 ± 0.428	94.66 ± 0.494 ^a^	105.33 ± 0.557 ^ab^	117.00 ± 0.577 ^abc^	140.16 ± 0.600 ^abcd^
Low density lipoprotein (LDL) (mg/dL)	40.00 ± 0.577	83.16 ± 0.600 ^a^	85.00 ± 0.365 ^a^	117.33 ± 0.760 ^abc^	143.66 ± 1.021 ^abcd^
High density lipoprotein (HDL) (mg/dL)	40.00 ± 0.577	30.66 ± 0.666 ^a^	26.33 ± 0.494 ^ab^	21.00 ± 0.365 ^abc^	18.00 ± 0.365 ^abcd^

Values are expressed as means ± SE. Statistical significance was tested using ANOVA followed by post hoc Tukey’s HSD multiple comparison test. ^a^ The mean values are significantly different in comparison with the control group at *p* ≤ 0.05. ^b^ The mean values are significantly different in comparison with the MSG 0.8 group at *p* ≤ 0.05. ^c^ The mean values are significantly different in comparison with the MSG 1 group at *p* ≤ 0.05. ^d^ The mean values are significantly different in comparison with the MSG 2 group at *p* ≤ 0.05.

**Table 4:** Effects of different doses of monosodium glutamate on oxidative stress and antioxidant parameters in the liver of male rats.

Liver Parameters	Experimental Groups				
	Control	MSG 0.8	MSG 1	MSG 2	MSG 3
Malondialdehyde (MDA) (nmole/g. tissue)	6.38± 0.047	7.55 ± 0.076 ^a^	18.63 ± 0.417 ^ab^	31.16 ± 0.477 ^abc^	38.83 ± 0.477 ^abcd^
Nitric oxide (NO) (µmole/g. tissue)	9.00 ± 0.365	22.00 ± 0.577 ^a^	28.50 ± 0.428 ^ab^	38.66 ± 1.782 ^abc^	42.50 ± 0.763 ^abc^
Glutathione (GSH) (mmole/g. tissue)	48.50 ± 0.428	39.50 ± 0.428 ^a^	33.00 ± 0.577 ^ab^	20.50 ± 0.428 ^abc^	17.00 ± 0.365 ^abcd^
Super oxide dismutase (SOD) (U/g. tissue)	76.50 ± 0.428	63.50 ± 0.763 ^a^	48.50 ± 0.763 ^ab^	43.83 ± 0.600 ^abc^	34.66 ± 0.666 ^abcd^
Catalase (CAT) (U/g. tissue)	54.50 ± 0.428	46.50 ± 0.763 ^a^	42.00 ± 0.577 ^ab^	31.00 ± 0.365 ^abc^	20.33 ± 0.760 ^abcd^
Glutathione-S-transferase (GST) (U/g. protein)	44.16 ± 0.600	32.33 ± 0.760 ^a^	27.50 ± 0.428 ^ab^	18.50 ± 0.428 ^abc^	11.16 ± 0.477 ^abcd^

Values are expressed as means ± SE. Statistically significance was tested using ANOVA followed by post hoc Tukey’s HSD multiple comparison test. ^a^ The mean values are significantly different in comparison with the control group at *p* ≤ 0.05. ^b^ The mean values are significantly different in comparison with the MSG 0.8 group at *p* ≤ 0.05. ^c^ The mean values are significantly different in comparison with the MSG 1 group at *p* ≤ 0.05. ^d^ The mean values are significantly different in comparison with the MSG 2 group at *p* ≤ 0.05.

**Table 5:** Effects of different doses of monosodium glutamate on oxidative stress and antioxidant parameters in the kidney of male rats.

Kidney Parameters	Experimental Groups				
	Control	MSG 0.8	MSG 1	MSG 2	MSG 3
Malondialdehyde (MDA) (nmole/g. tissue)	4.80± 0.036	8.01 ± 0.365 ^a^	19.16 ± 0.477 ^ab^	31.33 ± 0.557 ^abc^	42.83 ± 0.945 ^abcd^
Nitric oxide (NO) (µmole/g. tissue)	2.98 ± 0.087	15.16 ± 0.477 ^a^	22.33 ± 0.760 ^ab^	29.66 ± 0.557 ^abc^	35.33 ± 0.666 ^abcd^
Glutathione (GSH) (mmole/g. tissue)	48.50 ± 0.428	42.50 ± 0.619 ^a^	30.00 ± 0.365 ^ab^	24.33 ± 0.714 ^abc^	23.33 ± 0.714 ^abc^
Super oxide dismutase (SOD) (U/g. tissue)	67.33 ± 0.494	52.00 ± 0.577 ^a^	35.33 ± 0.494 ^ab^	25.83 ± 0.703 ^abc^	20.33 ± 0.494 ^abcd^
Catalase (CAT) (U/g. tissue)	37.00 ± 0.577	31.16 ± 0.477 ^a^	24.00 ± 0.856 ^ab^	18.00 ± 0.577 ^abc^	11.83 ± 0.600 ^abcd^
Glutathione-S-transferase (GST) (U/g. protein)	43.33 ± 0.494	30.66 ± 0.557 ^a^	27.33 ± 0.494 ^ab^	18.00 ± 0.365 ^abc^	12.83 ± 0.600 ^abcd^

Values are expressed as means ± SE. Statistically significance was tested using ANOVA followed by post hoc Tukey’s HSD multiple comparison test. ^a^ The mean values are significantly different in comparison with the control group at *p* ≤ 0.05. ^b^ The mean values are significantly different in comparison with the MSG 0.8 group at *p* ≤ 0.05. ^c^ The mean values are significantly different in comparison with the MSG 1 group at *p* ≤ 0.05. ^d^ The mean values are significantly different in comparison with the MSG 2 group at *p* ≤ 0.05.

**Table 6:** Effects of different doses of monosodium glutamate on oxidative stress and antioxidant parameters in the heart of male rats.

Heart Parameters	Experimental Groups				
	Control	MSG 0.8	MSG 1	MSG 2	MSG 3
Malondialdehyde (MDA) (nmole/g. tissue)	9.00 ± 0.365	22.33 ± 0.802 ^a^	28.33 ± 0.666 ^ab^	36.50 ± 0.763 ^abc^	52.50 ± 0.763 ^abcd^
Nitric oxide (NO) (µmole/g. tissue)	8.31 ± 0.300	17.50 ± 0.428 ^a^	24.66 ± 0.421 ^ab^	33.50 ± 0.921 ^abc^	40.16 ± 0.477 ^abcd^
Glutathione (GSH) (mmole/g. tissue)	49.00 ± 0.365	43.50 ± 0.763 ^a^	28.50 ± 0.428 ^ab^	23.16 ± 0.600 ^abc^	17.16 ± 0.477 ^abcd^
Super oxide dismutase (SOD) (U/g. tissue)	75.00 ± 0.577	50.50 ± 0.428 ^a^	39.83 ± 0.477 ^ab^	34.00 ± 0.577 ^abc^	18.33 ± 0.494 ^abcd^
Catalase (CAT) (U/g. tissue)	43.50 ± 0.763	35.33 ± 0.666 ^a^	22.33 ± 0.494 ^ab^	19.16 ± 0.477 ^abc^	14.00 ± 0.577 ^abcd^
Glutathione-S-transferase (GST) (U/g. protein)	48.00 ± 0.428	37.16 ± 0.477 ^a^	22.33 ± 0.666 ^ab^	19.00 ± 0.577 ^abc^	10.00 ± 0.365 ^abcd^

Values are expressed as means ± SE. Statistically significance was tested using ANOVA followed by post hoc Tukey’s HSD multiple comparison test. ^a^ The mean values are significantly different in comparison with the control group at *p* ≤ 0.05. ^b^ The mean values are significantly different in comparison with the MSG 0.8 group at *p* ≤ 0.05. ^c^ The mean values are significantly different in comparison with the MSG 1 group at *p* ≤ 0.05. ^d^ The mean values are significantly different in comparison with the MSG 2 group at *p* ≤ 0.05.

## Data Availability

The authors confirm that the data supporting the findings of this study are available within the article.
